# *Lachnoanaerobaculum* gen. nov., a new genus in the *Lachnospiraceae*: characterization of *Lachnoanaerobaculum umeaense* gen. nov., sp. nov., isolated from the human small intestine, and *Lachnoanaerobaculum orale* sp. nov., isolated from saliva, and reclassification of *Eubacterium saburreum* (Prévot 1966) Holdeman and Moore 1970 as *Lachnoanaerobaculum saburreum* comb. nov.

**DOI:** 10.1099/ijs.0.033613-0

**Published:** 2012-11

**Authors:** Maria E. Hedberg, Edward R. B. Moore, Liselott Svensson-Stadler, Per Hörstedt, Vladimir Baranov, Olle Hernell, Sun Nyunt Wai, Sten Hammarström, Marie-Louise Hammarström

**Affiliations:** 1 Department of Clinical Microbiology, Immunology, Umeå University, Umeå SE-901 87, Sweden; 2 CCUG – Culture Collection University of Gothenburg, Department of Clinical Bacteriology, Sahlgrenska University Hospital, Göteborg SE-413 45, Sweden; 3 Department of Infectious Disease, Sahlgrenska Academy of the University of Gothenburg, Göteborg SE-405 30, Sweden; 4 Department of Medical Biosciences, Pathology, Umeå University, Umeå SE-901 87, Sweden; 5 Department of Clinical Microbiology, Clinical Immunology, Umeå University, Umeå SE-901 87, Sweden; 6 Department of Clinical Sciences, Pediatrics, Umeå University, Umeå SE-901 87, Sweden; 7 Department of Molecular Biology, Umeå University, Umeå SE-901 87, Sweden

## Abstract

Two novel obligately anaerobic, Gram-stain-positive, saccharolytic and non-proteolytic spore-forming bacilli (strains CD3 : 22^T^ and N1^T^) are described. Strain CD3 : 22^T^ was isolated from a biopsy of the small intestine of a child with coeliac disease, and strain N1^T^ from the saliva of a healthy young man. The cells of both strains were observed to be filamentous, approximately 5 to >20 µm long, some of them curving and with swellings. The novel organisms produced H_2_S, NH_3_, butyric acid and acetic acid as major metabolic end products. Phylogenetic analyses, based on comparative 16S rRNA gene sequencing, revealed close relationships (98 % sequence similarity) between the two isolates, as well as the type strain of *Eubacterium saburreum* and four other *Lachnospiraceae* bacterium-/*E. saburreum*-like organisms. This group of bacteria were clearly different from any of the 19 known genera in the family *Lachnospiraceae*. While *Eubacterium* species are reported to be non-spore-forming, reanalysis of *E. saburreum* CCUG 28089^T^ confirmed that the bacterium is indeed able to form spores. Based on 16S rRNA gene sequencing, phenotypic and biochemical properties, strains CD3 : 22^T^ and N1^T^ represent novel species of a new and distinct genus, named *Lachnoanaerobaculum* gen. nov., in the family *Lachnospiraceae* [within the order *Clostridiales*, class *Clostridia*, phylum *Firmicutes*]. Strain CD3 : 22^T^ ( = CCUG 58757^T^  = DSM 23576^T^) is the type strain of the type species, *Lachnoanaerobaculum umeaense* gen. nov., sp. nov., of the proposed new genus. Strain N1^T^ ( = CCUG 60305^T^ = DSM 24553^T^) is the type strain of *Lachnoanaerobaculum orale* sp. nov. Moreover, *Eubacterium saburreum* is reclassified as *Lachnoanaerobaculum saburreum* comb. nov. (type strain CCUG 28089^T^  = ATCC 33271^T^  = CIP 105341^T^  = DSM 3986^T^  = JCM 11021^T^  = VPI 11763^T^).

Coeliac disease (CD) in children has features analogous to those of an infectious disease. Between 1985 and 1996, the incidence of childhood CD in Sweden among children younger than 2 years was four times higher than it was before or after (Ivarsson *et al.*, 2000). We have studied the microbial flora in biopsies from the jejunal mucosa of children born during the so-called ‘Swedish epidemic’ period and compared the microflora with those of control patients and CD children born after the epidemic period using scanning electron microscopy (SEM), cultivation-based analyses and 16S rRNA gene sequencing (Forsberg *et al.*, 2004; Ou *et al.*, 2009). We found that the microbiota of children with CD born during the Swedish epidemic had different compositions from those of children with CD born after the epidemic or those of controls, with a significant enrichment of rod-shaped bacteria of the *Clostridiales*, *Prevotella* and *Actinomyces*. Any or all of these bacteria may contribute to the aetiology or pathogenesis of CD in children. One of these rod-shaped bacteria was a spore-forming bacterium initially believed to belong to the clostridia (Ou *et al.*, 2009). The phylogenetic relationship of this *Clostridiales* sp. strain CD3 : 22 revealed that it was more closely related (98 % 16S rRNA gene sequence similarity) to the type strain of *Eubacterium saburreum* (CCUG 28089^T^  = JCM 11021^T^  = ATCC 33271^T^) and to several isolates and clones of the *Lachnospiraceae* than to the so-called ‘true’ clostridia belonging to cluster I of the subphylum *Clostridium* (Collins *et al.*, 1994). However, eubacteria have been described to be non-spore-forming (Holdeman *et al.*, 1977). The known habitat of *E. saburreum* is the oral cavity, where it has been isolated from dental plaque (L. V. Holdeman; type strain ATCC 33271^T^) and from the tongue dorsum (Tyrrell *et al.*, 2003).

This study describes the phenotypic and genotypic characterization of strain CD3 : 22^T^, strain N1^T^, a previously unreported, anaerobic oral strain, and the type strain of *E. saburreum*, CCUG 28089^T^. Additionally, we describe the phylogenetic relationships between the three isolates and other members of the family *Lachnospiraceae* based upon comparative 16S rRNA gene sequence analyses.

Strain CD3 : 22^T^ was isolated from a biopsy of the proximal small intestine of a girl with CD born in 1995, i.e. during the Swedish CD epidemic. She was on a gluten-free diet when the biopsy was taken at the Department of Paediatrics, Umeå University Hospital, Umeå, in 2007. The biopsy was weighed, homogenized and serially tenfold diluted in fastidious anaerobe broth medium (Lab M) and immediately plated onto selective and non-selective agar media. Strain N1^T^ was isolated from the saliva of a healthy young man in 1998 at the Karolinska Institute, Karolinska University Hospital, Huddinge, Stockholm, Sweden. *E. saburreum* CCUG 28089^T^, initially isolated from human dental plaque, was obtained from the Culture Collection University of Gothenburg (CCUG), Sweden. Pure cultures of the three strains grew well on blood agar plates [Columbia or Brucella agar base (BBL) supplemented with 5 % horse blood], on chocolate agar plates and in Brucella broth, under an anaerobic atmosphere (10 % H_2_, 5 % CO_2_ in N_2_) at 37 °C. Vitamin K and haemin were not required for growth and were not added to the media.

Colony morphology and presumptive identification tests by diagnostic discs (Jousimies-Somer *et al.*, 2002) were examined on blood agar plates after incubation for 2–3 days. Strains CD3 : 22^T^ and N1^T^ and *E. saburreum* CCUG 28089^T^ are all strictly anaerobic; they did not grow in the presence of oxygen. However, all three strains survived exposure to air for more than 12 h.

When the bacterial strains were cultured on a blood agar medium for 3 days, individual colonies of strain CD3 : 22^T^ were 1–3 mm in diameter, flat, spreading and circular with erose edges. The colonies were speckled and the centre of the colonies had a more compact appearance than the outer parts and, when viewed by stereomicroscope, they were sometimes multi-coloured, with pink, green, white and grey areas (Fig. S1, available in IJSEM Online). Colonies of strain N1^T^ had a similar size and speckled structure to those of CD3 : 22^T^ but with edges more rhizoid and with sometimes pyramidal centres, making the colony morphology clostridium-like. The colony morphology of *E. saburreum* CCUG 28089^T^ was also similar to that of CD3 : 22^T^, although the colonies lacked the compact appearance at the centre observed for CD3 : 22^T^ (Fig. S1).

The morphology of bacterial cells was investigated by light microscopy after Gram-staining, dark-field microscopy, SEM and transmission electron microscopy (TEM). Cells of strains CD3 : 22^T^ and N1^T^ were filamentous, 5 to >20 µm long. Some of the cells were curved and with swellings; chain forms were occasionally observed (Fig. S2a). The cells of strain N1^T^ were frequently observed in aggregates. Cells of *E. saburreum* CCUG 28089^T^ were longer, more slender and did not display the swellings or spore-like structures observed in CD3 : 22^T^ and N1^T^. All three strains were easily decolorized during Gram-staining.

The presence of spores was studied by Gram-staining, a specific spore-staining test, using malachite green (Shaeffer and Fulton spore stain kit; Sigma), and TEM. Spore formation was enhanced by growing the bacteria in Brucella broth at 37 °C for more than 4 days. Gram-staining detected spore-like structures in strains CD3 : 22^T^ and N1^T^ (Fig. S2b) but not in *E. saburreum* CCUG 28089^T^. However, spores were detected in all three strains by spore-staining (Fig. S2c, d). To confirm the presence of spores, TEM was performed on cultures of CD3 : 22^T^ and *E. saburreum* CCUG 28089^T^, identifying spores inside the cells and extracellular spores in both cultures (Fig. S2e, f). Treatment of bacterial cells with heat (70 °C for 10 min) killed the spores of the novel strains as well as spores from spore-forming positive-control strains of *Clostridium perfringens* and *C. innocuum*. Spores of the novel strains were killed by 75 % ethanol treatment for 30 min, but not by 50 % ethanol, whereas the clostridial spores were resistant to 75 % ethanol treatment but not 85 %.

All three strains exhibited a temperature optimum for growth at 37 °C. The pH optima were 6.5–7.0 for CD3 : 22^T^ and N1^T^ and 7.0–7.5 for *E. saburreum* CCUG 28089^T^. Motility was not observed. None of the strains was haemolytic. All three strains produced H_2_S and NH_3_. Growth on glucose as the sole carbon source yielded the following volatile fatty acids: for strain CD3 : 22^T^, butyric acid and small amounts of acetic acid; for N1^T^ and *E. saburreum* CCUG 28089^T^, butyric acid, acetic acid, lactic acid and small amounts of succinic acid.

Strains CD3 : 22^T^ and N1^T^ and *E. saburreum* CCUG 28089^T^ were resistant to 10 µg colistin and susceptible to 5 µg vancomycin, 1 mg kanamycin, 5 µg metronidazole and 20 % bile. Susceptibilities to penicillin G (M.I.C. Evaluator Strips; Oxoid) were determined to be 0.06 mg l^−1^ for strain CD3 : 22^T^, 0.3 mg l^−1^ for N1^T^ and 0.004 mg l^−1^ for *E. saburreum* CCUG 28089^T^.

The nucleotide sequences of the 16S rRNA genes of strains CD3 : 22^T^ and N1^T^ were determined by primer walking, covering the gene, and by cloning and sequencing of PCR amplification fragments, also covering the gene (Sambrook *et al.*, 1989). Other 16S rRNA gene sequences for comparative analyses were retrieved from the NCBI sequence database (Sayers *et al.*, 2010). The 16S rRNA gene sequence of CD3 : 22^T^ was unusual in that it was longer (1594 bp) than most bacterial 16S rRNA genes. It contained an insert of 110 nt at position 1436 compared with the sequence of *E. saburreum* CCUG 28089^T^. The sequence outside the insert exhibited 98 % similarity to that of *E. saburreum* CCUG 28089^T^. The sequence of strain N1^T^ did not contain this insert and was 98 % identical to the sequences of the other two strains (for CD3 : 22^T^ outside the insert). Fig. 1 shows the phylogenetic tree reconstructed by the maximum composite likelihood model, using 16S rRNA gene sequences, to display the relationships between strains CD3 : 22^T^ and N1^T^, *E. saburreum* JCM 11021^T^ ( = CCUG 28089^T^), *E. saburreum*-like isolate C27KA (Dewhirst *et al.*, 2010), *Lachnospiraceae* bacterium isolate F0167 and two sequences from related uncharacterized, uncultivated bacteria, one member each of the 19 genera of the family *Lachnospiraceae*, three species belonging to *Clostridium* cluster XIVa and a member of *Clostridium* cluster I (*Clostridium butyricum* VPI 3266^T^). Strains CD3 : 22^T^ and N1^T^ and *E. saburreum* CCUG 28089^T^ and related bacteria form a separate group distinct from the 19 known genera in the family *Lachnospiraceae*. This phylogenetic group is also distinct from members of *Clostridium* clusters XIVa and I. Based on cluster analyses and 16S rRNA gene sequence similarities between CD3 : 22^T^, N1^T^ and *E. saburreum* CCUG 28089^T^, these bacteria can be proposed to represent a novel genus, for which the name *Lachnoanaerobaculum* gen. nov. is proposed.

**Fig. 1.**
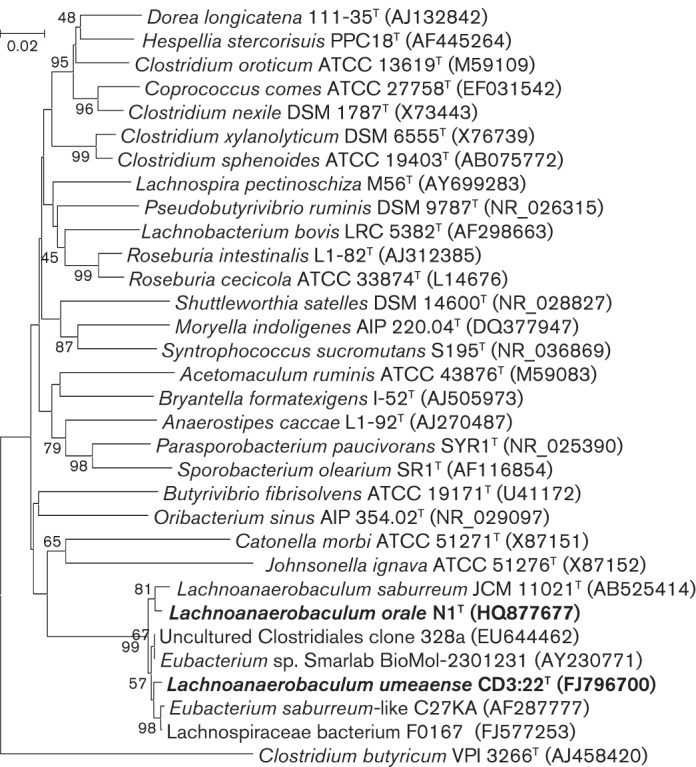
Phylogenetic tree based on 16S rRNA gene sequencing of members of *Lachnoanaerobaculum* gen. nov. and one species each of the other 19 recognized genera within the family *Lachnospiraceae* together with three remaining species of the genus *Clostridium* that are members of cluster XIVa. The 16S rRNA gene sequence of *Clostridium butyricum* VPI 3266^T^ served as an outgroup. Bar, 0.02 substitutions per nucleotide position.

The DNA G+C contents of the three strains were determined by HPLC as 35 mol% for strain CD3 : 22^T^, 38 mol% for strain N1^T^ and 37 mol% for *E. saburreum* CCUG 28089^T^.

Analyses of biochemical characteristics (Rapid ID 32A and API 20A; bioMérieux) showed that the three strains were saccharolytic and non-proteolytic (Table S1). However, they differed from each other in several respects. CD3 : 22^T^ and N1^T^ exhibited β-glucuronidase activity, while *E. saburreum* CCUG 28089^T^ did not. *E. saburreum* CCUG 28089^T^ and N1^T^ displayed indole production and pyroglutamic acid arylamidase activity, in contrast to CD3 : 22^T^. Acid production from carbohydrates was not detected in strain N1^T^ by the API 20A test, whereas strains CD3 : 22^T^ and *E. saburreum* CCUG 28089^T^ were more active. However, when grown in peptone yeast glucose broth, strain N1^T^ also formed volatile fatty acids. Strain N1^T^ was the only urease-positive strain. Another difference was that only CD3 : 22^T^ produced acid from mannose and raffinose. Finally, *E. saburreum* CCUG 28089^T^ displayed α-fucosidase activity, in contrast to the other two strains. Differentiating characteristics between the three strains are summarized in Table 1.

**Table 1.** Discriminating characteristics of strains CD3 : 22^T^ and N1^T^ and *E. saburreum* CCUG 28089^T^ Strains: 1, CD3 : 22^T^; 2, N1^T^; 3, *E. saburreum* CCUG 28089^T^. Data were obtained in this study.

Characteristic	1	2	3
DNA G+C content (mol%)	35.0	37.8	37.0
Enzyme activities			
Urease	−	+	−
α-Fucosidase	−	−	+
β-Glucuronidase	+	+	−
Pyroglutamic acid arylamidase	−	+	+
Indole production	−	+	+
Utilization of (API 20A):			
Mannose	+	−	−
Arabinose	+	−	+
Raffinose	+	−	−

Cell fatty acid methyl ester analyses were performed using a standardized protocol, similar to that of the MIDI Sherlock MIS system (described at http://www.ccug.se/pages/CFA_method_2008 and in File S1) (Sasser, 2001). Strains were grown anaerobically (85 % N_2_, 10 % H_2_, 5 % CO_2_) under the same conditions, using chocolate agar as a cultivation medium at 37 °C, and harvested after 40±2 h. Cellular fatty acids (CFAs) were extracted and saponified by mild alkaline methanolysis and the released fatty acids were methylated. CFAs were identified and quantified by GC (Hewlett Packard HP 5890). Retention times of CFA peaks were converted to equivalent chain-length (ECL) values and the relative amount (w/w) of each fatty acid was expressed as a percentage of the total fatty acids in the profile of the respective strain (Table S2). The major CFAs detected in strains CD3 : 22^T^, N1^T^ and *E. saburreum* CCUG 28089^T^ were C_14 : 0_, C_16 : 0_ and C_18 : 1_ω7*c* dimethylacetal (DMA). CFAs occurred in different relative amounts in the three strains. Thus, C_18 : 1_ω7*c* DMA constituted 16, 12 and 8 %, respectively, of the total CFAs for CD3 : 22^T^, N1^T^ and *E. saburreum* CCUG 28089^T^. There were also distinct differences in minor fatty acids (Table S2). The CFA profiles of the three strains differed, primarily, only in the relative amounts of any given CFA. The overall profiles of the three strains were distinctly similar. The isolation and characterization of more strains will enable more evidence to be presented as to whether the quantitative differences noted between the strains are characteristic of the individual strains or whether they represent species-level differences. Moreover, the fatty acid profiles of strains CD3 : 22^T^ and N1^T^ and *E. saburreum* CCUG 28089^T^ were distinct from the CFA compositions of type strains from the genera *Eubacterium* and *Clostridium* (Table S2). Overall, these data support the conclusion from the phylogenetic analyses that they indeed represent different bacterial species in a distinct and new genus.

Reciprocal DNA–DNA hybridization experiments were performed with strains CD3 : 22^T^ and N1^T^ and *E. saburreum* CCUG 28089^T^ at 60 °C for 16 h (Urdiain *et al.*, 2008). Low levels of genomic DNA–DNA relatedness (16.2–52.2 %) were observed between the three strains. The values were 52.1 and 38.7 % for CD3 : 22^T^ versus N1^T^, 16.2 and 40.2 % for N1^T^ versus *E. saburreum* CCUG 28089^T^ and 37.5 and 24.1 % for *E. saburreum* CCUG 28089^T^ versus CD3 : 22^T^. As the genomic DNA hybridization values were well below 70 %, the three strains can be confirmed to represent different species (Stackebrandt & Goebel, 1994).

We therefore conclude that strains CD3 : 22^T^ and N1^T^ represent novel species named *Lachnoanaerobaculum umeaense* gen. nov. and *Lachnoanaerobaculum orale* sp. nov., respectively. Finally, we propose that *Eubacterium saburreum* be reclassified as *Lachnoanaerobaculum saburreum* comb. nov.

## Description of *Lachnoanaerobaculum* gen. nov.

*Lachnoanaerobaculum* (Lach.no.an.ae.ro.ba′cu.lum. Gr. n. *lachnos* wool; Gr. pref. *an*- negating prefix; Gr. n. *aer* air; L. neut. n. *baculum* rod; N.L. neut. n. *Lachnoanaerobaculum* anaerobic rod forming woolly colonies).

Obligately anaerobic, Gram-stain-positive (easily decolorized and may appear as Gram-negative), spore-forming rods. Spores may be difficult to detect by Gram-staining. The cells are filamentous (5 to >20 µm long), sometimes curving and with swellings. Colony morphology on blood agar appears variably speckled and spreading; the edges are erose or rhizoid. Some colonies appear flat and others pyramidal. Temperature and pH optima for growth are 37 °C and pH 6.5–7.5. Saccharolytic and non-proteolytic, with butyric acid and acetic acid as major end products from glucose metabolism. Predominant CFAs are C_14 : 0_, C_16 : 0_ and C_18 : 1_ω7*c* DMA, accounting for 56–58 % of the total CFA profile. The G+C content of the DNA is 35–38 mol%. Strains have been isolated from the oral cavity, small intestine, blood and amniotic fluid (Han *et al.*, 2009) of humans. The type species is *Lachnoanaerobaculum umeaense*.

## Description of *Lachnoanaerobaculum umeaense* sp. nov.

*Lachnoanaerobaculum umeaense* (u.me.a.en′se. N.L. neut. n. *umeaense* of or pertaining to Umeå, referring to the discovery of the type strain at Umeå University).

Has the following properties in addition to those described for the genus. On blood agar, colonies are 1–3 mm in diameter, flat, spreading and circular with erose edges after 72 h at 37 °C. Colonies are non-haemolytic. Optimum growth at pH 6.5–7.0. Produces butyric acid, acetic acid, H_2_S and NH_3_. Acid is produced from glucose, mannose, xylose, arabinose, lactose, sucrose, maltose, trehalose, raffinose and melezitose. Activities for α-galactosidase, β-galactosidase, α-glucosidase, β-glucosidase and β-glucuronidase are observed but indole production and urease activity are not observed.

The type strain is CD3 : 22^T^ ( = CCUG 58757^T^  = DSM 23576^T^), isolated from a biopsy of the small intestine of a child with CD. The DNA G+C content of the type strain is 35 mol%.

## Description of *Lachnoanaerobaculum orale* sp. nov.

*Lachnoanaerobaculum orale* (o.ra′le. L. neut. adj. *orale* belonging to the mouth, referring to the isolation of the type strain from the oral cavity).

Has the following properties in addition to those described for the genus. Cells frequently occur in aggregates. On blood agar, colonies are 1.5–3 mm in diameter, spreading and circular with rhizoid edges and pyramidal centres after 72 h at 37 °C. Colonies are non-haemolytic. Optimum growth at pH 6.5–7.0. Produces butyric acid, acetic acid, lactic acid, indole, H_2_S and NH_3_. Acid production is not detected from any of 17 common mono- or oligosaccharides or sugar alcohols. Activity for α-galactosidase, β-galactosidase, α-glucosidase, β-glucosidase, β-glucuronidase and pyroglutamic acid arylamidase is observed. Urease activity is observed.

The type strain is N1^T^ ( = CCUG 60305^T^  = DSM 24553^T^), isolated from the saliva of a healthy young man. The DNA G+C content of the type strain is 38 mol%.

## Description of *Lachnoanaerobaculum saburreum* comb. nov.

*Laehnoanaerobaculum saburreum* (sa.bur′re.um. L. n. *saburra* sand; L.neut. stuff. -*eum* suffix used with the sense of belonging to; N.L. neut. adj. *saburreum* sandy). Basonym: *Eubacterium saburreum* (Prévot 1966) Holdeman and Moore 1970 (Approved Lists 1980).

Has the following properties in addition to those described for the genus. On blood agar, colonies are 1–2 mm in diameter, flat, spreading and circular with erose edges after 72 h at 37 °C. Colonies are non-haemolytic. Optimum growth at pH 7.0–7.5. Produces butyric acid, acetic acid, lactic acid, indole, H_2_S and NH_3_. Acid is produced from glucose, xylose, arabinose, lactose, sucrose, maltose, and trehalose. Activity for α-galactosidase, β-galactosidase, α-glucosidase, β-glucosidase, α-fucosidase and pyroglutamic acid arylamidase is observed.

The type strain is CCUG 28089^T^ ( = ATCC 33271^T^  = CIP 105341^T^  = DSM 3986^T^  = JCM 11021^T^  = VPI 11763^T^), originally isolated from dental plaque. The DNA G+C content of the type strain is 37 mol%.
